# Book Reviews

**Published:** 1814-01

**Authors:** 


### CRITICAL ANALYSIS
### OF RECENT PUBLICATIONS
IN THE
### DIFFERENT BRANCHES OF PHYSIC, SURGERY, AND MEDICAL PHILOSOPHY.

*Treatise on the History, Nature, and Treatment of Chin-cough; including a Variety of Cases and Dissections. To which is subjoined, an Inquiry into the relative Mortality of the principal Diseases of Children, and the Numbers who have died under ten Years of Age, in Glasgow, during the last thirty Years.* By ROBERT WATT, M.D. Member of the Faculty of Physicians and Surgeons of Glasgow, Member of the London Medical and Chirurgical Society, &c. and Lecturer on the Theory and on the Practice of Medicine in Glasgow. 8vo. pp. 392. Longman and Co. 1813.

WE have much pleasure in announcing to our readers the publication of this valuable work. The disease of which it treats is indeed familiar enough to our observation, but, like some others, it has not experienced that attention from practitioners, which its importance and fatality ought to have secured. Every family expects its visitation, and it has become a prevalent fashion to permit this malady to pursue its course with little interruption or assistance, unless such as is afforded by gossips, amulets, or domestic recipes. Is this abandonment of a most serious ailment to feeble, rash, and unauthorised hands, to be attributed to the inefficacy of medicine, or to the negligence and apathy of practitioners? We are disposed to inculpate the latter, and moreover to opine, that whenever the cure of a disease becomes intrusted to unprofessional hands, or that the medicines are supplied duly stamped, a newspaper advertisement, the sole guide and director, from a medicine warehouse, the source of such a sacrifice of understanding, must arise from the conduct of regular practitioners themselves. In the present instance, medical men have been forward to acknowledge that the disease depends upon a peculiar contagion, will run its course in spite of medicine, and the practice they have pursued has too frequently consisted of uncertain, trifling, and unscientific remedies. Medicines the most opposite in quality and effect, have been given upon empirical principles, which it ill becomes such practitioners to adopt and apply, as are for ever exclaiming against quackery, and denouncing its pernicious consequences.

Fatal

Fatal as hooping cough appears to have been from the earliest records of its appearance, Dr. Watt has been able to discover very few works of consequence upon the subject. He could not trace it in the writings of the Grecian, Roman, or Arabian physicians; while in Britain, and most parts of Europe, no period can be assigned in which the disease did not exist; in London it increased rapidly during the last century; and in Sweden, according to Dr. Rosenstein's account, chiefly taken from the bills of mortality, 43,393 children died of it between the years 1749 and 1764. In the year 1755, 5832 children were carried off by this distemper; and in the years when it has raged with less fatality, about 1700 or 2000 are lost by it.* In Glasgow, the scene of the author's observations, it has continued very uniform for the last thirty years, but rather on the increase. In some years the number of deaths is greatly more than in others, but on the whole it has run pretty nearly about five, or from that to five and a half per cent. of the whole deaths in the city. The greatest number of deaths by chincough in Glasgow, during the last thirty years, was in 1809, when they amounted to 259, or something more than eleven and a half per cent. of the whole deaths in the year. It is somewhat singular, that in the last twenty-five years, there have been only six single months in which some have not died of chincough; and in the last twelve months, not a month has elapsed without a death. Next to the small-pox formerly, and the measles now, chincough is the most fatal disease to which children are liable.

The author considers the disease as highly infectious, and arising from a specific contagion, the degree of which depends on the state of the atmosphere. There are very few instances recorded in which it is supposed to affect a person more than once. It chiefly occurs in infancy and childhood, though no age is exempt from it. From a table inserted, it appears that March is the most fatal month; and that fewer die in the months of July, August, and September, than in any other part of the year. It has also proved much more fatal in some years than in others.

The author thus describes the early symptoms:

"The chincough generally begins like a common cold, attended with more or less fever. The lips appear full, some degree of hoarseness and sneezing succeed, with a redness or turgescence of the face, and generally a watery irritating discharge from the eyes and nostrils. The patient has a greater disposition to sleep than usual, some difficulty of breathing or oppression about the chest, with a hard dry cough.

* On the Diseases of Children, and their Remedies, p. 194.

cough. The appetite is somewhat impaired, attended with a costive or irregular state of bowels. The patient complains more than usual of cold, particularly in the back and extremities. After some days, the reverse of this takes place, he then complains of thirst, and a sense of heat over the whole body, the skin feeling harsh and dry.

"During the continuance of these symptoms there is occasional headach. The tongue is a little whiter and fouler than usual. The pulse is somewhat accelerated, and now and then irregular.

"This febrile state, which attends the commencement of Chincough, resembles in many respects the fever which precedes the measles or the small-pox. In some cases it is so mild, and continues so short, as hardly to deserve notice; in others it is more severe and protracted.

"In the generality of cases, as the kinks begin to form, the catarrhal and febrile symptoms abate, and the patient in the intervals feels himself quite well; but in others the fever continues, the kinks are less regular, and in the intervals the patient is languid and oppressed. The tongue continues foul, and the appetite bad. The pulse in these cases is weak, excessively quick, and often irregular. The skin assumes a livid color, while the patient's flesh and strength decline rapidly. This is one of the worst forms of the disease, and is most apt to occur in the young and plethoric. It is often fatal, for the danger is not apparent, till it is too late for any remedy to be of service.

"It has been remarked, that the chincough generally begins like a common cold. In some instances it continues in this form throughout the whole course of the disease. 'I have had instances of a disease,' says Dr. Cullen, 'which though evidently arising from the chincough contagion, never put on any other form than that of a common catarrh.' * The same circumstance has occurred within my own observation; and Mr. Burns remarks, that 'in young children, even death may take place, although the disease never fully forms.' †

"*Description of the paroxysm.*—Commonly about the end of the second, or beginning of the third week, the symptoms undergo a very remarkable change. During the cough the expirations become extremely rapid and altogether involuntary. By the continuance of this process the air is suddenly expelled from the lungs, and the patient appears in danger of suffocation. At last he is relieved by a full and violent inspiration, generally accompanied by a stridulous noise, like the crowing of a cock, or the rapid passage of air through a narrow brazen tube. The convulsive expirations are again renewed, and the paroxysm, consisting of these two parts alternately, is continued till relief is obtained either by expectoration or vomiting. The matter rejected is generally a tough mucus, occasionally mixed with the contents of the stomach, and sometimes with particles of blood.

"This manner of terminating the paroxysm, however, happens only in the more favorable cases; in the more severe, the paroxysm ends in the complete exhaustion of the patient, without any discharge whatever.

* First Lines of the Practice of Physic, Section 1406.
† Principles of Midwifery, &c. p. 541, 2d Edit.

"The expirations during the kink resemble those of a common cold; but are much more rapid and violent. The inspirations, in most cases, bear a very strong resemblance to those of well-formed Croup. It is from the peculiar sound produced by the last of these processes that the disease has been termed *Hooping* cough. The *Hoop*, however, is in many cases completely wanting, and the patient feels no obstruction to the taking in a full breath. 'I have said,' observes Dr. Butter, 'that the Kinkcough is *generally* attended with a shrill inspiration; because it sometimes occurs without that symptom. Such a defect constitutes a variety of the disease, which may properly be denominated the *imperfect Kinkcough*.' *

"It may perhaps be owing to this difference in the nature of the kinks, that in the Bills of Mortality for London, particularly about a century ago, some of the deaths are arranged under the head of Chincough, and others under that of Hooping cough. Even in later periods, from the manner in which the two terms are introduced, one would naturally conclude, that two distinct diseases were meant. Be that as it may, with regard to the circumstance of having or wanting the *Hoop*, I have remarked a very striking difference, which deserves to be noticed. In one set of patients, we have the *Hoop* very long and distinct; but in these the expirations are less rapid and violent. The patient seems to gasp and strain, rather than cough. The face is dark-colored, the eyes are turned up, and there is generally a tendency to fainting or strangulation. The patients affected in this way are generally the very young and the plethoric. In the other set of patients, the expirations are rapid, and so very violent, that, to use the common expression, they look as if they would burst. The face in such cases is of a bright scarlet color, the eyes fiery, and as if they would start from their sockets; but the inspirations are performed as soon, and apparently with as much ease, as if nothing were the matter. Cases of this kind occur in the older and more robust.

"These bursting kinks, as the disease advances, become less violent. The expirations produce a sort of barking noise, and the cough is less confined to regular paroxysms. Sometimes the expirations are of this kind from the beginning, and continue so throughout the whole course of the disease. Mr. Burns perhaps alludes to this form, when he compares the cough to 'the chattering of a monkey quickly repeated.' †

"Dr. Gregory, sen. has noticed a very curious variety of the disease, of which Dr. Kirkland gives the following account: 'Duos notavit casus celeberrimus noster Præceptor Gregorius, quibus non continuo tussiendi paroxysmo sonora inspiratio, sed loco inspirationis syncope superveniebat, et, aliquot horæ sexagesimis post syncopen interjectis, demum subsequebatur, solito more, inspiratio et alter tussiendi paroxysmus.' ‡

"When the kinks are of the Hooping kind, the face, as I have

* Treatise on Kinkcough, p. 3.

† Principles of Midwifery, &c. 2d Edit. p. 541.

‡ Dissertatio Inauguralis de Pertussi, p. 3. 1772.

already already remarked, becomes swollen, and of a dark purple color, the veins of the head and neck are distended as if ready to burst, and the patient gasps, and has the appearance of sudden suffocation. If he remains long in this situation without making any noise, he is said to have taken a *dumb kink.* In those violent efforts the extremities become cold, a sweat breaks out on the forehead and temples, and even blood is frequently forced from the nose, mouth, ears, and eyes. In many cases, the patient is actually suffocated, falls into a faint, or is seized with convulsions. When the inspirations are long and very difficult, the patient is said to have fallen into the *back draughts.* This symptom is generally regarded as little better than the *dumb kink,* both of them showing the disease to be of a very unfavorable nature.

"We have seen that in children the form of the kink is very different; in adults we meet with a similar variety; but the hoop seldom or never occurs. The bronchial system, at this period, is perhaps incapable of taking on that morbid state on which it depends. In two young women between twenty and thirty, the kinks were very severe and frequent. The face was flushed, and the whole body much agitated; but there was no apparent difficulty in the inspirations. In the lady's case, who had this disease along with her children, the kinks were of the same description, but accompanied with a most violent headach. These were the only adults I remember to have seen under the disease.

"Dr. Heberden remarks, that 'adults are, as it were, overpowered at once upon the access of the fit, so that they fall down instantly, as in an apoplexy, but they very soon come to themselves; this is a distinguishing symptom of the disease in those who are grown up.' *

"Dr. Butter gives the case of a woman of thirty, the mother of several children, and at the time in a state of pregnancy. He does not mention any affection of the head among the symptoms, but remarks, that 'sometimes the fit was so severe as to threaten suffocation. She sometimes expectorated a white thick phlegm, and then the fit was short. A severe fit was always attended with a clear ropy expectoration. She complained also of pain in her right side, which sometimes shot through to the other, and sometimes downward, so as to imitate labour pains.'† The disease was subdued in a short time by the use of hemlock and other remedies. The patient was afterwards delivered of a healthy child, and had a good recovery."

The fits vary much in frequency and severity, and recur at irregular intervals. The appetite is various, at times voracious, at others impaired. The author considers a great desire for drink a bad sign, and deems a free indulgence hurtful. He thinks "a tea-spoonful or two will quench the thirst as well as a full draught, while it weakens and oppresses the stomach much less." In this particular we can-

* Commentaries on the History and Cure of Diseases, p. 434.

† Treatise on the Kinkcough, p. 61.

not unite with him in opinion, having never witnessed any unfavorable circumstance from allowing a child a moderate portion of any aqueous beverage. Dr. Watt had previously remarked, that nothing brings on a fit of coughing more readily than "fretting the patient." Now, we can hardly conceive any thing more likely to fret a child parched with thirst, and exhausted with the violence of former paroxysms, than snatching the honeyed cup from his lips before he had well tasted the refreshing beverage.

Some judicious remarks occur on the fever which is present in Hooping cough, and which in some instances nearly resembles Typhus. The author points out three states of fever as deserving of particular notice: the first is that anomalous febrile state, which accompanies, more or less, even the mildest forms of the disease. It seems to partake more of the inflammatory than the typhoid type. The pulse is variable, easily accelerated, rather hard, and at times irregular. The second state is typhoid. "In this state there is nothing so remarkable as the quickness of the pulse. From being full and moderately slow, it passes at once to almost insuperable frequency, and is often so weak as hardly to be felt." The third state of the fever is decidedly inflammatory. "The pulse is not so quick, but it is full and hard, attended with considerable difficulty of breathing, occasional pains in the chest, increased heat, loaded tongue, and all the other signs of pneumonic inflammation.

The author has less to observe on the state of respiration in Hooping cough, than we think requisite; we have no objection to his drawing upon Dr. Darwin, for the practical remarks of that writer are always valuable; but in a work evidently considered by its author as the only complete account of the disease of which it treats, we did expect to be favored with some original observations upon this very important function; as it is, the following paragraph comprises the sum of the author's information.

"From the state of the pulse, and the state of the breathing, we can draw more certain conclusions, as to the nature and severity of the disease, than from any other circumstance whatever. *I have never met with almost any case,* where either the one or the other was much affected, where there was not very considerable danger; and none where the two symptoms were conjoined, that did not prove fatal, unless opposed by the most vigorous treatment."

We are not clear that our conception of the author's statement, "I never met with almost any case," is correct, but we suppose him to mean that he *has* met with cases in which either the pulse or the breathing were much affected, without "there being considerable danger,"

Among

Among the unfavorable symptoms, Dr. Watt has noticed the urine deficient in quantity, of a high color, becoming turbid on standing, the patient having a frequent desire to void it, while the quantity discharged is very small; the skin uncommonly dry and insensible to the touch; a pale leucophlegmatic appearance; skin, especially of the face and extremities, of a livid and dark color; œdema of the feet and hands; nostrils dry, and itchy; cutaneous eruption, if any, disappearing.

The reader will meet with some valuable remarks on the termination of the disease; the diagnostic symptoms; and symptoms of Bronchitis, which we have not space to detail. The account of the opinion of early writers on hooping cough, will also prove interesting to the medical scholar. The length to which this article has already extended, will not admit our citing any of the author's cases; but we shall avail ourselves of his account of the dissections, because they are much less frequent than in most other diseases. We are all of us well acquainted with the symptoms of hooping cough, yet few of us have investigated the appearances after death.

The first dissection was of the author's own son, aged six years.

"The body was opened by Mr. Robert Limon, a pupil of mine, sixty-five hours after death, and the following notes were taken at the time by my friend, Mr. William Couper, surgeon, of this city.

"'The lungs appeared to fill the whole cavity of the thorax more completely than they generally do.

"'The anterior surface of the lungs appeared as if covered with whitish-colored flat tubercles, (not unlike bad confluent small-pox,) as if some white fluid had been effused under the covering pleura. On making some incisions into the substance of the lungs, the cells were found filled with a whitish purulent-looking mucus, with only a small admixture of air. The cells immediately under the investing pleura, thus filled, were the cause of the external appearance of the lungs above described. Some very small portions of the lungs appeared to have the cells less full of this mucus than the rest, the external surface of these portions having a more natural appearance.

"'The posterior surface of the lungs had a brighter appearance than the anterior, having more the look of recent inflammation.

"'The inside of the trachea from the thyroid gland downwards, to an inch below the bifurcation, had its inner surface smeared over with the same kind of mucus, as that filling the cells of the lungs. Beyond this its branches appeared completely filled with the same mucus.

"'The whole inside of the trachea, and its ramifications, was painted with red vessels, appearing to have been the seat of recent inflammation.'

"At

"At this time it was thought unnecessary to carry the investigation farther; but next day my friend Mr. Muir, of Paisley, expressed a wish to ascertain the state of the stomach and bowels. In consequence of this the abdomen was opened, and the following notes were taken by Mr. Muir respecting the contents. It should be remarked, that even at this late period, there were hardly any signs of putrefaction.

"'On laying aside the integuments, the intestines and stomach presented themselves in a very white or bleached-like appearance. There was a long patch of inflammation on part of the small intestines, and beside it, another smaller mark of recent inflammation. A similar appearance was found on one part of the great arch of the colon, in which there was no fæces, but a little thin greenish-colored fluid. The mesocolon was wholly very dark colored, nearly approaching to gangrene. On turning up the stomach, the under and external surface was as if painted with a brown color like new-burnt coffee in appearance, and there was little of it free from traces of this color. It was as if laid on with long strokes of a brush half an inch broad. Some of them were, however, narrower and shorter than others. On laying open the stomach, the internal surface had numerous red streaks, the marks of recent inflammation; there was an universal crust of lymphatic exudation, and much of it was collected in the upper part, owing to the position of the viscus, when the body was examined.'"

Dissection 2.—Janet Watt, sister to the above, aged four years and a half. Her body was opened twenty-five hours after death, and the following notes taken by Dr. Graham:

"'The skin is generally of a livid color, and very dark in some places, an appearance which it had a tendency to assume some time before death. There were no signs of putrefaction. On opening the chest, the lungs were found of a purple color, irregularly interspersed with whitish spots slightly elevated, which were found to be owing to a mixture of mucus and air under the pleura. There were no adhesions between the pleuræ, but the lungs had not collapsed nearly so much as usual. On the convex surface of the right lung a very small quantity of tough purulent-looking matter was observed, which seemed to have escaped by one or two small openings in the pleura, whether the consequence of rupture or erosion could not be determined. Similar matter was found interspersed through the substance of the lungs, but there was no where any cyst containing it. The lungs felt knotty, and uncommonly firm in some places; but on cutting into them no tubercles, such as are met with in phthisis, could be detected. In some places though their bulk was increased, they were firm, and sunk in water; in others they sunk from being collapsed, though not indurated. The pleura costalis seemed rather more vascular than common. The trachea was slightly inflamed on its internal surface, and covered with a quantity of frothy mucus, intermixed with small portions of a purulent-like matter, which increased in quantity downwards, till it nearly plugged up the smaller ramifications. Before opening the pericardium, it seemed to contain a larger quantity quantity of fluid than is usually observed, but it having been accidentally punctured, any fluid it might have contained escaped, without being noticed.

" 'The abdominal viscera seemed sound. The gall-bladder was full of dark-colored, thick, viscid bile.' The head was not examined, but from the nature of the symptoms, there was little reason to expect disease in that quarter."

Dissection 3.—Margaret Beaton, aged three years and three months.

" The body was opened forty hours after death, in presence of Mr. Russel, several of his pupils, and myself. The skin was more livid than usual, but there were no signs of putrefaction.

" 'On opening the chest, the lungs appeared to have collapsed about as much as usual. Their anterior surface was more irregular than is generally found, and thickly mottled with a whitish ash color. These whitish patches occupied about two thirds or more of the whole surface of the lungs, and were slightly elevated above the rest. Pressure on any part of the lungs produced or increased these whitish spots, around where the pressure was made, apparently by forcing along the air and mucus under the investing pleura. Almost the whole of the right lung had nothing of a cellular feel, but, as Mr. Russel observed, it felt like a piece of spleen or liver. On cutting into the substance of the lungs, in various places, the air cells appeared to be filled with a viscid semipurulent mucus, which oozed out in great abundance. A considerable margin along the lower edge of each lung was of a bright red color, felt solid to the touch, and when cut off, sunk readily in water.

" 'On laying open the upper part of the trachea, its internal surface was of a reddish floculent appearance, but without any inflammatory exudation. This inflammatory appearance increased as we proceeded downwards, till, in the more minute ramifications of the bronchiæ, the whole surface was of a bright red color, as if painted; but still there was little or none of that purulent-like mucus, usually found on inflamed secreting membranes. As far as we could trace these passages, the ingress and egress of the air appeared to be unobstructed.

" 'There were numerous adhesions between the right lung and the pleura costalis of that side; but these appeared to have been of some standing, or perhaps had no connection with the present disease. They were more probably the effects of a pneumonic attack, which she had about a year ago, and since which she has always had more or less dyspnœa, particularly on any considerable exertion.

" 'The contents of the abdomen appeared to be sound, with the exception of about a foot of the ilium, which was very vascular, and of a red color. Part of the mesocolon appeared also to be somewhat redder and more vascular than usual.

" 'On removing the skull cap, the dura mater adhered to it very firmly, but did not show any signs of disease. The veins over the surface of the brain were very turgid, but perhaps not more so than we we usually find them in young subjects. There was no water in the ventricles, and the other parts of the brain appeared to be perfectly sound.' "

Dissection 4.—Miss K. aged four years and a half.

" The body was opened twenty-five hours after death by Mr. Limon, in presence of Mr. Russel and myself, and the following notes were taken.

" ' The body appears very much emaciated. The skin is darker than usual, and stained here and there with livid spots. Left arm and leg œdematous. A place on the breast and neck, where a blister had been recently applied, of a dark livid color. No signs of putrefaction in other respects.

" ' On opening the thorax, the lower part of the right lung was found to adhere firmly and extensively to the pleura costalis of that side. This portion of the lung seemed to be enlarged, and occupied fully that portion of the chest. It was of a whitish color, had lost all appearance of cellular structure, and on being pressed with the finger, it felt hard and gristly in some parts and soft and pulpy in others.

" ' On removing the adhesions, a large abscess was discovered, ramifying through this part of the lung in all directions, and partly filled with purulent matter. The immediate parietes of the abscess were of a dense cheesy consistence, and varied from a quarter of an inch to an inch in thickness. Beyond this solid portion, the whole substance of the right lung was interspersed with tubercles, exactly similar to those met with in ordinary cases of pulmonary consumption. Many of these tubercles were smaller than peas, and some of them as large as hazel nuts. Some of them were slightly, others more extensively inflamed, and many of them were in a state of suppuration.

" ' The whole surface of this lung was covered with a layer of inflammatory exudation, and in many places the investing pleura appeared to be very much eroded and destroyed.

" ' The left lung was much more collapsed than the right, and exhibited those irregularities on its surface, and the same mottled appearances described in former cases. On examining its substance between the finger and thumb, it was found to have the same tubercular feel as the right, and much more solid than natural.

" ' Some parts had a peculiar reddish purple color, and felt more uniform in their texture than the rest. The color of these portions seemed to depend on the quantity of blood contained in them. When cut off, they sunk readily in water.

" ' On cutting into this lung, the number of tubercles was nearly as great as in the right, and were as much diversified in point of size, but fewer of them had arrived at a state of suppuration. On this side there were no adhesions, and the investing pleura did not appear to have suffered from inflammation as in the right.

" ' The mediastinum appeared to have suffered in the first instance from inflammation, and latterly its cellular portion had become œdematous. On pulling up the sternum, part of it adhered to its inner surface, and part of it remained floating between the lungs like a quantity of jelly. The pericardium contained a much larger quantity of fluid than usual, but the heart itself appeared to be sound.

" 'The whole of the larynx and trachea were then removed out of the body and examined. The glottis and epiglottis were slightly inflamed. The internal surface of the trachea was also a little more vascular than usual, and was covered with the natural mucus. On proceeding farther down, the vascularity seemed to increase, but even on entering the bronchiæ, it hardly deserved the name of inflammation. At no place as far as it was traced, could any thing like a lymphatic coat be discovered. Here and there we could perceive some particles of purulent matter, but these had evidently arisen from the abscess in the lungs.

" 'On opening the abdomen, the liver was found much enlarged, extending into the left hypochondrium, and downwards into the umbilical region. Its surface was of a whitish ash or clay color, resembling boiled liver. The structure did not appear to be much changed, but the whole substance felt somewhat denser than usual. The gall bladder was full of a thin light-colored bile.

" 'The spleen was also enlarged, and its surface was rough and vascular, as if it had suffered from inflammation.

" 'The stomach was very much contracted, being little more capacious than the rest of the intestines. The internal coats were much corrugated, but no signs of disease. It contained a small quantity of fluid like thin gruel.

" 'The intestines were here and there of a dark color, but this seemed to depend entirely on these portions having lain in a more depending posture than the rest. Owing to the want of time, the head was not examined. But we had little reason to expect any morbid appearances in that quarter.' "

For the treatment of this disease, Dr. Watt has enumerated all the most favorite and approved remedies of the writers, which he has with much industry consulted; and though we do not find any thing novel in this part of the subject, we think he has fairly weighed the merit of the several modes of cure which he has noticed, has shown what combinations of medicines are most likely to succeed, and pointed out others which have less beneficial effects than have usually been attributed to them. This part of the work occupies 130 pages.

An appendix is subjoined, which contains a very interesting inquiry into the relative mortality of the principal diseases of children, and the numbers who have died under ten years of age in Glasgow, during the last thirty years. We shall content ourselves with selecting a few extracts.

"Taking an average of several years, I found that more than a half of the human species died before they were ten years of age, and that of this half more than a third died of the small-pox, so that nearly a fifth part of all that were born alive perished by this dreadful malady."

Since the introduction of vaccination, the proportion of deaths from small-pox diminished in an astonishing degree; but on the other hand the fatality of measles was very much increased. "In the small-pox we have the deaths reduced to nearly a fifth of what they were twenty-five years ago; in the same period, the deaths by measles have increased more than eleven times." Thus, as the small-pox disappears, measles advance to keep up the usual waste of human life. The fact with regard to Glasgow is indisputable, and the author thinks that measles must in some degree be modified by small-pox. "That the measles (he remarks) should have been thus modified by the small-pox, is rendered highly probable by various analogical facts. The manner in which the vaccine disease prevents the small-pox is quite in point, and when it has failed in preventing them altogether, it has rendered them so mild, as not to be attended with the smallest danger. May not small-pox have a similar effect with regard to measles?"

This is an important subject for inquiry, but we have little apprehension as to the result. We are somewhat surprised indeed that a writer possessed of so much acuteness and spirit of investigation, as is Dr. Watt, should have so readily embraced a conclusion founded on analogy; that, admitting the facts, he should have taken so little trouble to find other causes than that which he has alleged. Neither do we think his argument much strengthened by his quotation from Dr. Woolcombe, who makes Providence create children for the mere purpose of destroying them. "Since disease," says that physician, "is one of the appointed checks to excessive population, and the plan of Providence in the creation of human life, requires the termination of the existence of one third of its creatures, before they have attained the age of two years, it may be doubted whether the annihilation of so efficient an instrument as small-pox can be admitted without the substitution of some other equally destructive malady." It is not for such insignificant beings as reviewers to canvass the particular designs of Providence in the creation of man; but without flying at so high a quarry, we may be allowed to question the propriety of calling in the Deity upon every occasion, to solve difficulties which a little attention on our own part might possibly explain. If men by debauchery unfit themselves for generating a healthy progeny, are enfeebled by the operation of poverty and unwholesome employments, or match themselves to unfit help-mates, are they to call in question the mercy of God, and accuse him of those consequences which are the natural result of their own misconduct?

conduct? Epidemical disorders, indeed, seem to be beyond the control of man; but we well know that his mode of living and peculiar circumstances have a very modifying effect upon them, and even these may be explained without invoking Apollo.

We have not had an opportunity of comparing the London bills of mortality with those of Glasgow during the period Dr. Watt describes; but as far as extensive public practice in a wide district, and inquiry from physicians in great practice in other neighbourhoods, can inform us, the fatality of measles does not appear to be increased in London since the introduction of vaccination.

It seems the number of still-born children at Glasgow has increased something more than a per cent. and a half on the whole deaths. Dr. Watt, however, does not accuse either small-pox or cow-pox of occasioning this misfortune, but attributes it to "the introduction of particular manufactures, by which immense numbers of women are employed in public works or confined to sedentary employments, by which the general health and vigor of the system must be materially impaired." "From this cause we may calculate not only on a greater number of dead-born children, but on a more puny offspring in general, and consequently on a greater number of deaths in infancy." So say we, and may not measles be influenced by such causes? The only fatal cases which have occurred to us, and these are very few, were precisely under such circumstances, the puny offspring of mechanics, dwelling in close unwholesome apartments.

In concluding, we hope that the spirit of inquiry on the subject will go forth, and that we shall shortly have accounts from different parts of the kingdom. If we have differed in opinion from the ingenious author, it has been, we trust, from the same desire for truth, and to encourage that ardor for investigation in others, which he possesses in an eminent degree. Esse enim contemplationem veritatis, omni operum utilitate et magnitudine digniorem et celsiorem.

---

MEDICAL TRANSACTIONS, *published by the* COLLEGE *of* PHYSICIANS *in* LONDON. Vol. IV. pp. 415.—Longman and Co. 1813.

WE congratulate the profession on the appearance of this volume, which indeed has been somewhat long in collecting, for the first papers it contains were read upwards of seven years ago. Still the subjects are interesting, and being the productions of some of the leading physicians in London, have strong claim to notice. In a collection of cases and practical observations, there is little scope for criticism; the captious, indeed, may always find something to cavil at, the laborious critic to display his dulness, and the smart writer his wit; but on such an occasion, in our opinion, the proper mode of proceeding is to gratify our readers with offering them the most valuable papers in the volume.

Dr. M. Baillie has contributed two cases. The first is of a boy seven years of age, who had hydrocephalus, and in whom some of the bones of the skull, once firmly united, were, in the progress of the disease, separated to a considerable distance from each other. This was the only circumstance deserving remark in the case. Dr. Baillie regards it as a very rare occurrence, none of his medical friends having met with such an instance; and the doctor states that he has not found an example of it in any of the books which he has consulted. He admits, however, that want of time prevented his looking into as many books as he could wish. Dr. Baillie certainly can employ his time to more advantage than in turning over books; but we think he might have found similar instances without losing many of his golden moments. We merely opened two; the first was Tulpius, who was very eminent in his day, and has left a valuable work behind him: in the fifth edition of his *Observationes Medicæ*, published at Leyden, 1716, lib. 1, cap. xxiv. p. 45, is the following case. "Puer quinquennis, dissectus in valetudinario publico, congesserat in capite tantum liquoris, ut vitiatâ naturali figurâ, tantoperè inter se descisserent dissoluta ossa: ut dilatata ipsorum compages, haud parùm discreparet, a virili magnitudine." If it be objected that in this subject the bones had been partly absorbed by the action of so much pressure from within, perhaps the following instances from Bonetus, in p. 305 of the first volume of the folio edition published at Geneva, 1679, may be regarded as more in point, although the age of the patients either is not given, or is younger than in Dr. Baillie's case. "Imo quandoque cerebrum universum diffluat, juxta Zacutum, aut ex cerebro irruens aqua *suturas* dissolvat." Ex Fontano, Pract. lib. 1, cap. iii.

Another case of a boy aged two years, "cui per hydrocephalum superiores omnes et legitimæ suturæ, præter unam lambdoideam, quæ parum hiabat, ita erant diductæ, ut aqua incumbens cerebro per diaphanam cutem transpareret."

In page 307, the same author explains the progress of the disorder in separating the sutures in young subjects, for in the adult it cannot be done. "In provecta enim ætate, ac senibus, suturæ non tantum arctissime junguntur, verum etiam non raro cranium plane coalescit, ut vestigia tantum cerebrum supersint. Hinc pueris aqua, priusquam cerebrum premat, ventriculos distendit, hac augescente, etiam suturas, suturas, non ægre cedentes divellit, tandem etiam membranas expandit, imo et ipsum cerebrum, membranarum in modum ductile; unde caput ad eam molem, quæ in hydrocephalis conspicitur, excresscit: hinc aqua capaci spatio conclusa, minus premit et distendit. In senibus autem quorum cranium durum ac compactum, si aqua quotidie incrementa sumat, defectu spatii; obvia quæque comprimit, atque eousque donec arctatis omnibus spirituum animalium viis, apoplexia illos abripiat."

Jacobus Fontanus, in pract. lib. 1, cap. iii. gives the dissection of a boy, in which the sutures were severed to the extent of four fingers. "Distabant autem suturæ fere digitis à se invicem."

We now return to Dr. Baillie's case:

"The great peculiarity in the progress of this boy's disease is the separation of some of the bones of the scull from each other, which had once been firmly united, in consequence of the accumulation of water in the ventricles of his brain. It may very naturally be enquired, whether there was any peculiarity in the sutures of this boy's scull, by which some of its bones, once firmly united, had, by gradual distension from within, been separated from each other. In examining the edges of the two parietal bones at the sagittal suture, the processes of union appeared to be more simple in their form, and fewer in number, than is usual in children of the same age. The same circumstances were observed in examining the edges of the frontal and parietal bones at the coronal suture. Had the processes at the edges of these bones been more numerous, and more irregular in their form, it is very difficult to conceive how they could have been separated in the progress of this disease, from each other. If the boy had been a few years older, the same effect most probably would not have taken place; and in an adult, the bones of whose scull were united by the usual strength of sutures, it would have been impossible.

The edges of the frontal and parietal bones were thinner than is usual at the same period of life; but this effect had been probably produced by the gradual pressure of the brain against these bones, in consequence of the accumulation of water in the ventricles. Had the patient lived still longer, they would probably have become still thinner, through the continued influence of the same cause keeping up an increased action in the absorbent vessels of these bones.

II. *Of some uncommon Symptoms which occurred in a Case of Hydrocephalus Internus.* By MATTHEW BAILLIE, M.D. F.R.S.

"A Gentleman, aged fifty-six, was seized with symptoms of compression of the brain, on the 9th of February, 1805, and became became completely paralytic on the right side, upon the 11th of the same month. By this attack he lost his recollection of the words of his own language, except a very few, which he pronounced with the greatest distinctness, exhibiting none of that thickness in his pronunciation which is so common in paralytic patients. These words, *yes*, *yes*, *no*, *no*, *Mr. Reed*, *yesterday*, were employed upon all occasions, with a great variety of tone, to express pleasure and displeasure, joy and sorrow, to explain the circumstances of his disorder, and to give directions about what he wanted. He did not seem to be aware that these words were not the proper ones to express his meaning, for he often betrayed impatience when he was not understood, and was not mortified at repeating so often the same words: other words he could pronounce distinctly, but he hardly ever did it, except in repeating some word that any person in his presence had just uttered. His countenance expressed a full share of understanding, and he seemed to comprehend whatever was said or done by others in his presence. His eyes appeared in a natural state; the pupils were not dilated, and his sight was perfect. He sometimes expressed a feeling of pain in the upper part of his head, by putting his left hand there. His skin was sometimes a little hot: his pulse was sometimes between ninety and a hundred, was often of the natural frequency, but was never very slow, nor irregular.

"During the first four or five months, nothing remarkable was observed in the paralytic limbs; but about six months before the death of the patient, the right foot was contracted involuntarily inwards, and the right hand was bent upwards and forwards upon the fore-arm. The fingers soon afterwards were contracted into the palm of the hand, and the fore-arm was bent upon the arm. This state of the upper extremity was sometimes attended with pain, and varied a little in the degree of contraction at different times. During the last weeks of his life, the right arm acquired so much rigidity in this posture, as to resemble in some degree the permanent attitude of a Fakir in Hindostan. Soon after the right foot was contracted inwards, the right leg was bent back upon the high, and the right thigh was bent upwards and forwards upon the trunk of the body. This however was not in the same degree as the bending of the right arm which we have just described. Within a few weeks of his death, there was also some degree of involuntary contraction in the left leg and thigh, but there was none in the left arm. About two months also before the patient died, the right thigh and leg became much swelled, and there were even appearances which denoted a tendency to mortification; but these soon vanished, and the the swelling gradually in a great measure subsided. During this uncommon disease, the bowels were for the most part rather costive: the urine was generally in the natural quantity, sometimes a little scanty, but was never remarkably deficient. In the course of so long and severe an illness, the temper of the patient was sometimes irritable, but it was often very calm, and he exhibited great kindness of disposition, which made a part of his natural character. On the 6th of January, 1806, after an illness of eleven months, he died, and, for a few days previous to his death, he was almost in a constant state of drowsiness.

"At the beginning of his attack he was directed to lose blood by the arm, was cupped, and had a blister applied to his head. Some purgative medicines were ordered for him, which were continued in a milder form during the greater part of his illness. When he was languid, he was directed to take some mild tonic, and when his irritation was considerable, it was appeased by an opiate. For five or six weeks he took four grains of the Pilulæ Hydrargyri every night, with the view of diminishing by absorption the matter which was evidently compressing the brain; but without any advantage. Very little benefit indeed was expected from medicine from the first attack of the complaint; but from time to time it relieved some troublesome symptoms as they occurred.

"Some of the circumstances of this disease were so singular, that I had much more than a common curiosity to examine the brain after death. My patient's relations very liberally acceded to my wishes upon this subject, and the brain was examined in my presence on the 8th of January, 1806, viz. two days after he died. The membranes of the brain and its substance were perfectly natural in their appearance. The blood-vessels were not too much loaded with blood, and there was no appearance of blood having been extravasated in any part of the substance of the brain. The lateral ventricles of the brain however were found to contain rather more than six ounces of water. Besides this, there was no other diseased appearance, except that the left vertebral artery was enlarged in its size, and its coats had become opaque. The result of this examination very much surprised me. Although symptoms of pressure upon the brain were very strongly marked, yet none had occurred during any part of the disease, which usually denote the accumulation of water in the ventricles."

III. *A singular Case of Stricture and Thickening of the Ileum, by* Dr. CHARLES COMBE. *Communicated by* WILLIAM SAUNDERS, M.D. F.R.S.

"William Payne Georges, esq. of a very nervous and delicate habit, had been for many years troubled with flatulency and and complaints in the bowels, attended with costiveness and a quick pulse, which generally beat between 90 and 100 in a minute. Towards the end of September, 1805, he had an irregular intermittent fever. From this time all his former complaints rapidly increased. He especially complained of wind and great costiveness, and about two hours and a half or three hours after eating, of excessive pain in the bowels. So intense was this pain, that for the last three weeks he refused taking any solid food, and subsisted entirely on jelly, broth, milk, &c. and these only in very small quantities, always dreading the pain that succeeded even this kind of food.

" When he had a natural stool, it was passed in very small indurated lumps, either separate or attached together, bearing some resemblance to sheep's dung. Besides the above complaints, he had been for several years troubled with an uncommon pulsation of the aorta about the region of the loins, which was not only perceptible to himself internally, but even by the hand applied externally, upon the umbilical region. This inconvenience continued to increase to the time of his death, which took place early on Sunday morning, February the 9th, 1806, at which time he was more emaciated than any person we had ever witnessed.

" On the next day the body was opened by Dr. Combe in the presence of Dr. Saunders, and the following appearances were observed.

" There was not any fat between the skin aud the muscles of the abdomen, or about any of the parts of the integuments. The epiploon was entirely wasted, and there was not the least appearance of fat about the mesentery or kidneys. The liver, pancreas, spleen, and kidneys, were in a sound state. The stomach, though rather smaller than usual, was in a natural state, as was the duodenum, the jejunum, and the upper part of the ileum. The lower part of the ileum, as far as the colon, was contracted, for the space of three feet, to the size of a turkey's quill. The colon had three constrictions, one about three inches long at the distance of seven inches from the cœcum, a second about one inch long at the distance of four inches from the former, and a third not quite half an inch long at the distance of three inches from the last.

" In consequence of the constriction of the ileum, and the total want of fat in the abdomen, there was nothing to support the colon in its natural situation. It had, therefore, fallen, and lay irregularly on the small intestines, and consequently there was no appearance of what is called the arch of the colon. Wherever the intestines were constricted, the

 coats coats were very much thickened, and exhibited an appearance of inflammation; while the blood-vessels, passing through the mesentery to the intestines, were enlarged. The aorta was in a perfectly natural state.

"Though the lacteals are distributed to the whole of the intestines, they are most numerous about the ileum, and we believe nearly all the chyle is absorbed before the food reaches the colon. In proportion as the coats of the intestines thicken, the absorbent power of the lacteals is diminished, and in the constricted part of the ileum in this case we believe the absorbent power of the lacteals was totally destroyed. Hence arose the general marasmus. From the ileum which lay upon the aorta, being inflamed, and therefore more irritable, and from the absence of fat which would lessen the apparent action of the aorta, Mr. Georges became very sensible of the pulsation, and suffered considerably from it. It became perceptible to the hand in the same manner as the pulse in the wrist, for so greatly was he emaciated that the finger and thumb might be placed on each side of the aorta, and be strongly sensible of the lateral pulsation. The time at which Mr. Georges perceived the great pain and uneasiness to commence after eating, corresponds with the period at which the food would naturally arrive at the beginning of the constriction of the ileum. The figure of the stools we apprehend arose from the constrictions of the colon."

IV. *Cases of Tetanus in consequence of Wounds; evincing the utility of relaxant medicines, and more especially of the pulvis ipecacuanhæ compositus in large and repeated doses.* By J. LATHAM, M.D. F.R.S.

This is a very satisfactory paper, and fully establishes the good effect of opium and James's powder; and especially of pulvis ipecacuanhæ compositus, in some cases of Tetanus, as the following instances will prove.

"The first case which more especially called my attention to this subject, occurred to Mr. Hawkins, of Croydon, who stopped me in the street to enquire where he might find our common friend Mr. Long, as he wished to inform him that the patient whom they had seen together a few days before, for a bruise about the elbow, had been seized with locked-jaw. In our conversation respecting it, he asked me if I knew how to cure it: I told him that I had never yet been successful in a single instance, but that in the first case which should occur to me, I had determined to push boldly the relaxant plan, and give large and repeated doses of James's powder. He without hesitation stated his determination to try the method suggested, but doubted whether he ought not to persist in the use of opium; which however he had hitherto been giving without any manifest advantage. It was agreed that he should still continue the usual remedy; the powder and the opium were given *alternately;* the result was favorable, for in the course of a few weeks, I had the satisfaction of knowing that the patient had perfectly recovered.

"The next case occurred to me in St. Bartholomew's Hospital: the patient's hand had been bruised dreadfully, and one of his fingers crushed to pieces. A locked-jaw with almost universal tetanus succeeded; and such was the violence of the muscular spasm every ten minutes, that it seemed very likely soon to put a period to his sufferings. I directed for him five grains of James's powder every four hours and for several days had the satisfaction of finding that the muscular rigidity diminished, and that the convulsive motions of the system were less painful and less frequent. So much benefit seemed manifestly to have been produced, that I was firmly persuaded that a cure was about to be effected, and at the desire of the pupils, who were very anxious about the case, I made it the subject of a clinical lecture, in the course which I was then delivering at the hospital. The publicity of the case became, as I then strongly suspected, the cause of our disappointment; for the pupils propagating the favorable report which the apparent advantage derived from the powder had enabled me to make, a constant succession of visitors unfortunately interrupted that progress which the patient seemed to be making towards a complete recovery. His spasms returned with violence, and the jaw which had become considerably relaxed, was now much more rigid. In consultation it was determined to remove the finger, and to give opium in large doses: the original plan was then forsaken, and in the course of the next day a dreadful recurrence of spasm, which had indeed throughout the night violently tortured him, put an end to his existence."

The third case, after exhibiting very favorable symptoms from the use of the medicine, terminated rather unexpectedly, by the patient being seized suddenly with a succession of spasms which destroyed him in a few hours. Having succeeded in one case out of three, Dr. Latham was determined not to relinquish the principle, but in future proposed to try the pulvis ipecacuanhæ compositus. The first case which occurred after this resolution, was at St. Bartholomew's Hospital, the particulars of which we shall transcribe.

"John Triggs, a middle-sized strong-built man, aged twenty-five, by employment a groom, had the misfortune to have a fall from his horse on Tuesday, the 23d of October,

179S.

1798. It was not discovered that he had received any other injury than a slight wound on the outer ancle of the right leg, which was healing very kindly under the care of Mr. Blicke, in St. Bartholomew's Hospital, into which he had been, immediately after the accident, admitted.

"On the 30th of October, the wound was almost closed, when he was suddenly seized with a rigid sensation about the muscles of his neck, which, as he described it, held his head firmly down, as if bound with cords: his lower jaw became affixed to the upper with little or no power of extending it, and his whole body, and especially his wounded leg, was affected by spasms. In the more severe attacks he felt a violent pain under the sternum, leading upwards from the scrobiculus cordis, in the direction of the ensiform cartilage.

"At the beginning his pulse beat 120 strokes in the minute, he sweated profusely, and numerous miliary eruptions appeared about the wrists. On the following day, October 31st, he was visited for the first time by Dr. Latham, who ordered ten grains of the Pulvis Ipecacuanhæ compositus every four hours with Ol. Ricini occasionally: the wound was at the same time dressed with a common bread and milk poultice, and he was kept on the milk-diet of the hospital.

"On making frequent enquiries concerning his feelings, he said he thought himself gradually becoming better, his pains and spasms having abated, and his jaws admitting of a thin slice of bread soaked in milk. In a few days he could put the point of his thumb between his teeth, though not without exerting some force; and a regular amendment continued till the 12th of November, when the pains under the sternum returned, the affected limb became spasmodic, with twitchings in various parts of his body and profuse sweatings. These unfavorable symptoms were supposed to have arisen from his imprudent omission of the Oleum Ricini, for they were much alleviated by a proper evacuation of the bowels. On the 13th he was better, but there being a continuance of the spasms in a slight degree, the doctor shortened the intervals between the administration of the medicines from every four to every three hours. On the 14th, a perceptible advantage was observed from the increase of the quantity of the pulv. ipec. comp. within the last twenty-four hours. He was now more comfortable, his jaws admitting of wider distension, and allowing him to take larger quantities of food. In this manner he proceeded, daily improving, until the 18th, when the poultice was thrown aside, and the wound was again attempted to be healed. Appearances continuing to be favorable, the time of administering his medicines was altered on the 20th from every three to every four hours. On the 23d he was so greatly recovered that he was ordered to take decoct. cinchonæ ʒxiv. cum tinct. cinchonæ ʒj. every six hours, and the time of giving the pulvis ipec. compositus was altered from every four to every six hours.

"His appetite grew so keen, and he gained strength so rapidly, that on the 4th of December the milk-diet was changed to that of meat, and he was allowed to get out of bed.

"Every symptom having disappeared, and the wound being healed, he was discharged from the hospital on the 13th of December.

"It is worthy of remark, that during the whole time of taking the pulvis ipecacuanhæ compositus he felt no unusual inclination to sleep, although in every ten grains of it he was swallowing one grain of pure opium, and that too every four hours, and sometimes oftener. He complained of no other inconvenience from it than a continued sweating, with a trifling degree of debility from being kept so long in bed."

In another case in which this remedy proved beneficial, the symptoms were less violent. The last case, which we give intire, is very memorable. "At the very time," says Dr. Latham, "I was arranging the histories of the foregoing cases, and considering the probable efficacy of Pulvis Ipecacuanhæ compositus in the cure of this disease, Mr. Eaton, an apothecary in Holborn, wished me to see his relation who was his shopman, and who from cutting his hand by corking a bottle about three weeks before, had been laboring for three days under the very worst possible state of Tetanus. For his better accommodation, Mr. Eaton had removed his patient to Somers-Town, who was then attended by Mr. Ford of Golden-square. The account which he gave me of the young man's sufferings was distressing in the extreme; and he added that, in his opinion, as well as in that of Mr. Ford, nothing was likely to serve him; but that for his own satisfaction, as a relation, he was desirous that I should see him. I took from my drawer the very cases above recited, and read them to him, under the idea of encouraging him to persevere with resolution in the plan which I proposed. He was accordingly ordered to give his patient ten grains of pulvis ipecacuanhæ compositus every two hours until I visited him, which I promised to do early in the morning. On seeing him according to my promise, I found him (as he expressed himself) not worse, but, as he thought, a little more comfortable: I therefore directed him to persevere in the use of the powder, which he continued regularly to take almost twenty days. During this time the spasms were occasionally very dreadful; for he was usually drawn into an irregular arch, his head and his feet forming its two extremities: his urine was often forced from him by the violence of the spasms, and his belly was frequently drawn inwards, and very obstinately constipated. Fully sensible of his situation, he not only persevered in the use of his medicine with astonishing resolution, but of his own accord took two or three times a-day ten or twelve drops of tincture of opium. Although his jaws were so locked together as scarcely to admit at any time the edge of a shilling, yet he contrived to get the medicine into his mouth, either by squeezing it, when made into a soft bolus, through the small aperture which his utmost efforts could make, or by taking it as occasion allowed him in a liquid form. The comparative degree of ease which he sometimes experienced encouraged him to go on, notwithstanding the sudden recurrence of the spasms, and the violence of them each time, which nearly hazarded his very existence. I purposely omit in my account of this case many circumstances, such as the regulating his bowels by clysters or by castor oil; the giving broths or light nutritious liquids; the allowing a little portion of wine diluted with water, when sudden faintness or deliquium supervened. Such arrangements of course were made as occasions required; but I more especially wish to lay stress upon the facts that the compound powder of ipecacuanha was never once omitted, as I believe, for more than a fortnight, that it never induced any remarkable drowsiness, that the perspiration was generally great, although not immoderate, and that nothing like sickness occurred until the 17th or 18th day, when he appeared to those about him suddenly to be giving way,—but on vomiting a little glairy fluid he found relief, and the alarm of his friends ceased. Until this time his amendment had been gradual; but after this sudden deliquium and sickness, and a further relief obtained by evacuating the bowels, his spasms seemed greatly to abate; and now for the first time he began to dislike the powder, which indeed did not longer seem very necessary, but which was nevertheless given in smaller doses and at greater intervals. He was now directed to take wine more freely, and to begin a course of bark in decoction with the tincture and aromatic confection. In a week from this time he rapidly recovered, and in a few weeks afterwards was enabled to attend to his business as usual.

"After two or three years, a piece of glass which had been gradually making its way, was extracted by Mr. Ford from between the thumb and first finger of the patient's hand. Mr. Ford, who saw this case in the first instance, and who kindly called occasionally upon the patient during the continuance of the complaint, declared to me, that of all the cases which he had seen (ten or twelve) this was by much the worst."

V. *Remarks on Tumors, which have occasionally been mistaken for Diseases of the Liver.* By J. LATHAM, M.D. F.R.S.

The affections which the author thinks are most likely to be confounded with diseases of the liver, are scirrhous pancreas and enlargement of the right ovarium. They occasion the symptom of diseased liver, by producing pressure on the biliary ducts. "I take it for granted (he observes) that nobody can easily mistake an *inflammation* of the liver: the pain in the organ (either acute when its investing membrane is affected, or obtuse when its parenchymatous substance suffers) and the febrile exacerbations regularly consequent thereupon, will sufficiently mark this disease: neither, if the liver can readily be felt, and if the usual icteric symptoms be present, can we be very much deceived as to the viscus affected; but when, perhaps for the first time, a patient is only conscious of pain, when his physician investigates his case by pressure about the right hypochondrium and scrobiculus cordis, when his appetite has gradually failed him, when he is troubled with eructations, and his stools have been clayey and adhesive, then I suspect that the liver is becoming inactive, notwithstanding the skin and eyes may yet be clear, and the urine scarcely tinged with bile. If pressure discovers a deeper-seated uneasiness under the scrobiculus cordis, but neither enlargement nor pain in the right hypochondrium; and if the patient tells me that although his appetite is not amiss, yet eructation and vomiting commonly follow his meals, I then suspect, notwithstanding the eyes and skin may be yellow, the urine full of bile, and the stools without any, that the pancreas alone may be the organ affected, which pressing upon the stomach produces nausea and sickness, and obstructing the passage of the bile through the hepatic ducts occasions every symptom of an *apparently* diseased liver. If, without any perceptible hardness of the liver, I observe the true characteristic yellowness of the eyes and skin and urine, notwithstanding pain should be acutely felt in the direction of the biliary ducts, yet if (the pain not remitting) the fæces should sometimes be well tinged with bile, and at other times be without any such appearance, I should suspect that the symptoms were produced by an indurated pancreas. If an acute pain is constantly felt in the direction of the biliary ducts, and strikes through to the back with that sort of sensation as if something something was affixing the liver thereto, whether the fæces be tinged with bile or not, a biliary concretion either in the ductus cysticus, or in the ductus communis choledochus, most probably produces it.

"But a diseased ovarium, more commonly than an enlarged pancreas, produces, by its pressure upon the biliary ducts, every symptom of indurated liver. The delicacy which is properly due, and is rightly observed in every disease of females, too often, however, hinders that examination which in its early stages would always lead us to a just distinction. We are told that there is a fulness in the right side: we see clearly that there are icteric symptoms, but we thence conclude too hastily that the disease is in the liver. In this as in the case of indurated pancreas, the appearances of the skin and urine and fæces will vary; and hence also we have at once a *presumptive* proof that the liver is not the *permanent* seat of the disease. We should therefore immediately solicit an examination, which will usually put the matter beyond all doubt; for, I believe, upon pretty strong pressure, the ovarium, when enlarged, will be traceable as ascending from a circumscribed base from below upwards, and the liver (as in all women, whether it be diseased or not, this organ may usually be felt) will be found by an equal pressure descending from above downwards; but between one viscus and the other, whether ovarium or liver, the same degree of pressure will, I think, always discover a sort of chasm or space, denoting a termination of the enlargement: *below*, therefore, the enlarged viscus must be the ovarium, *above* it will be the liver. I do not, however, mean to say that in all possible cases this mechanical sort of distinction must necessarily hold good; yet in the early stages of the disease it will never, I believe, mislead us, and in the later periods of it, with exact attention to collateral circumstances, it will seldom fail. I will not here mention any other tumors of the abdomen, as these are easily enough to be distinguished from enlargements of the liver: for such only which are manifestly attended with icteric symptoms, and are thence liable to be confounded with real hepatic disease, can regularly come within the scope of my present observations."

Upon the method of cure Dr. Latham does not presume to know more than his brethren; in these obscure diseases the reputation of the physician seems to depend more upon his ability in discriminating, than in his power of curing them.

*A full Analysis of all the other papers contained in this important Volume, will be given in our next.*

MEDICAL

